# Supplementary material for: A Mammalian Surface Display Platform to Optimize the Antigenicity of Viral Proteins for Vaccine Design
Source: bioRxiv. 2026 Jan 12:2026.01.09.698728. Preprint. [Version 2] doi: 10.64898/2026.01.09.698728 (PMC12871315; doi:10.64898/2026.01.09.698728)

bioRxiv preprint doi: https://doi.org/10.64898/2026.01.09.698728; this version posted January 12, 2026. The copyright holder for this preprint (which was not certified by peer review) is the author/funder, who has granted bioRxiv a license to display the preprint in perpetuity. It is made available under aCC-BY 4.0 International license.

Supplementary Figure 1

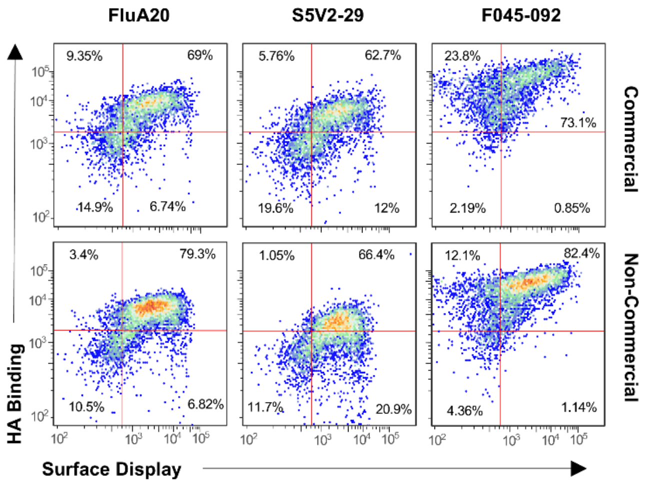

bioRxiv preprint doi: https://doi.org/10.64898/2026.01.09.698728; this version posted January 12, 2026. The copyright holder for this preprint (which was not certified by peer review) is the author/funder, who has granted bioRxiv a license to display the preprint in perpetuity. It is made available under aCC-BY 4.0 International license.

Supplementary Figure 2

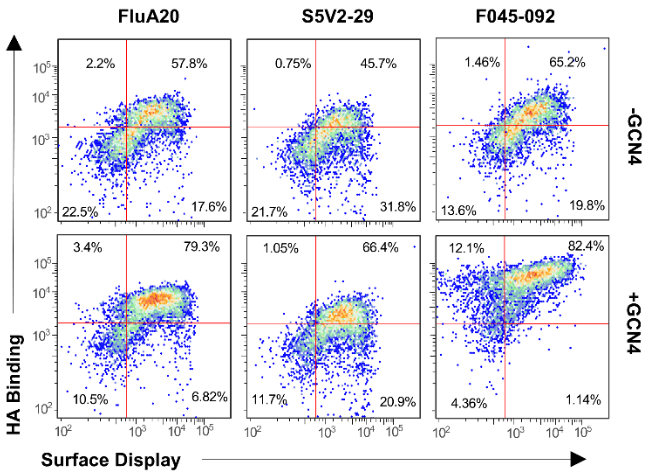

bioRxiv preprint doi: https://doi.org/10.64898/2026.01.09.698728; this version posted January 12, 2026. The copyright holder for this preprint (which was not certified by peer review) is the author/funder, who has granted bioRxiv a license to display the preprint in perpetuity. It is made available under aCC-BY 4.0 International license.

Supplementary Figure 3

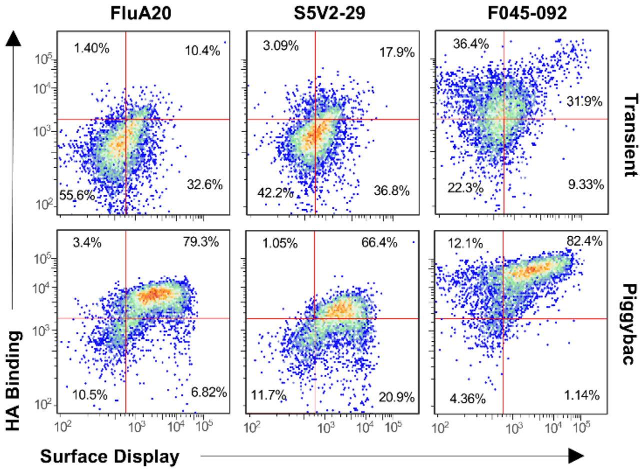

bioRxiv preprint doi: https://doi.org/10.64898/2026.01.09.698728; this version posted January 12, 2026. The copyright holder for this preprint (which was not certified by peer review) is the author/funder, who has granted bioRxiv a license to display the preprint in perpetuity. It is made available under aCC-BY 4.0 International license.

Supplementary Figure 4

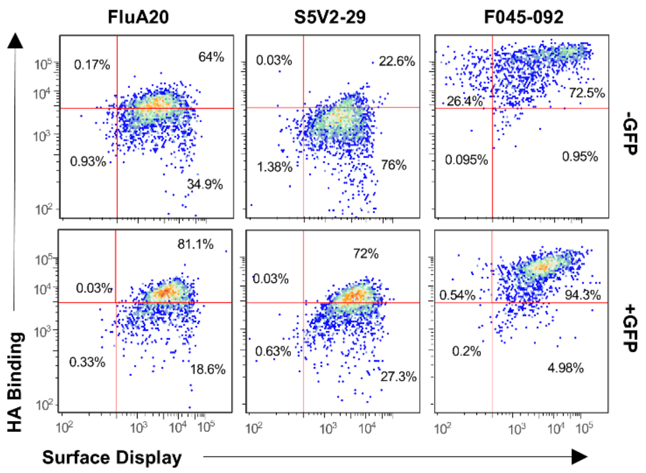

bioRxiv preprint doi: https://doi.org/10.64898/2026.01.09.698728; this version posted January 12, 2026. The copyright holder for this preprint (which was not certified by peer review) is the author/funder, who has granted bioRxiv a license to display the preprint in perpetuity. It is made available under aCC-BY 4.0 International license.

Supplementary Figure 5

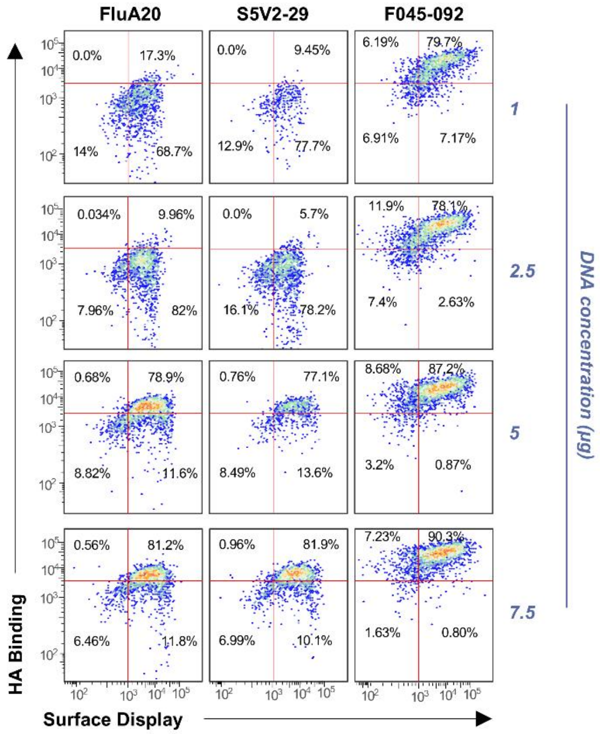

bioRxiv preprint doi: https://doi.org/10.64898/2026.01.09.698728; this version posted January 12, 2026. The copyright holder for this preprint (which was not certified by peer review) is the author/funder, who has granted bioRxiv a license to display the preprint in perpetuity. It is made available under aCC-BY 4.0 International license.

Supplementary Figure 6

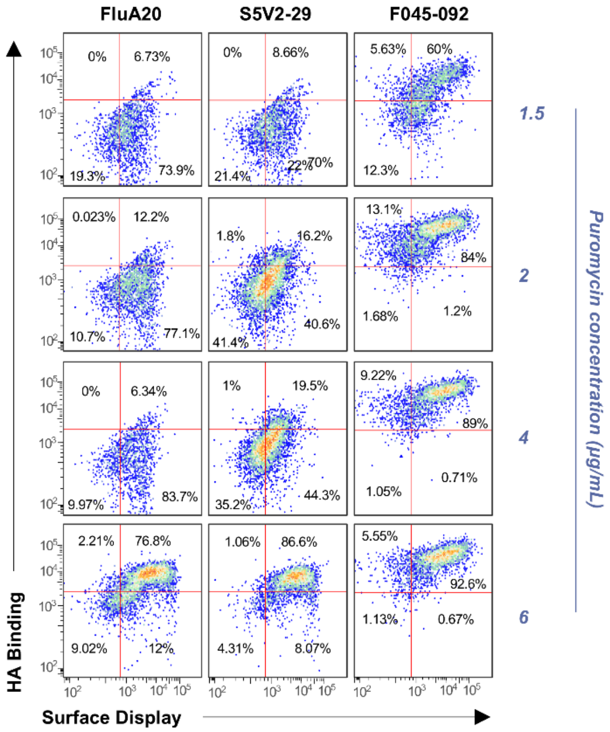

bioRxiv preprint doi: https://doi.org/10.64898/2026.01.09.698728; this version posted January 12, 2026. The copyright holder for this preprint (which was not certified by peer review) is the author/funder, who has granted bioRxiv a license to display the preprint in perpetuity. It is made available under aCC-BY 4.0 International license.

Supplementary Figure 7

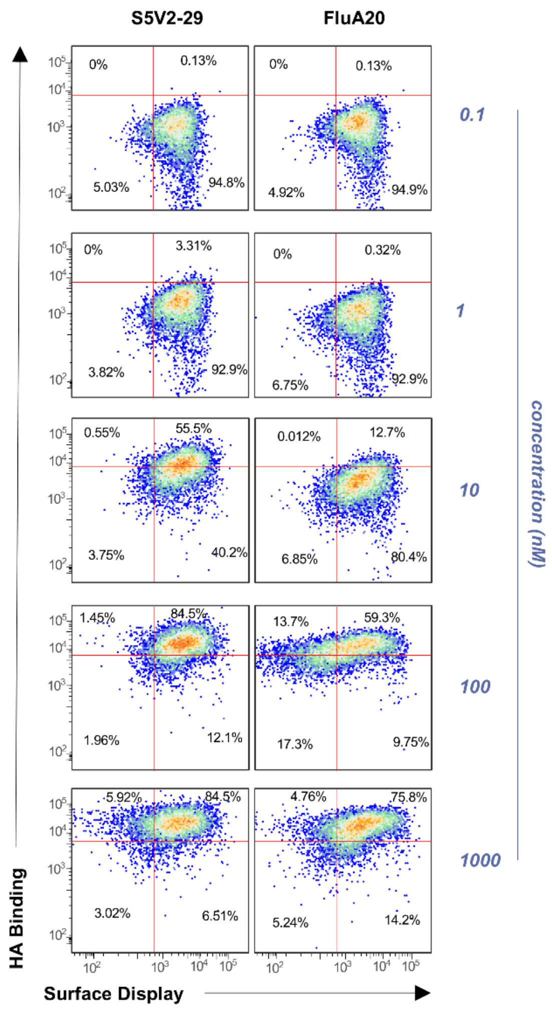

bioRxiv preprint doi: https://doi.org/10.64898/2026.01.09.698728; this version posted January 12, 2026. The copyright holder for this preprint (which was not certified by peer review) is the author/funder, who has granted bioRxiv a license to display the preprint in perpetuity. It is made available under aCC-BY 4.0 International license.

Supplementary Figure 8

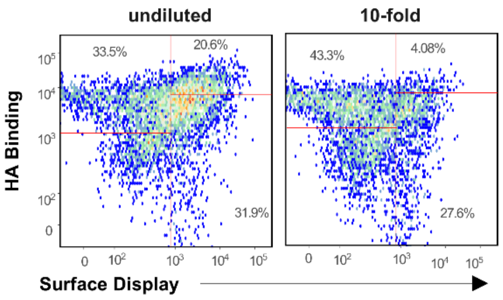

bioRxiv preprint doi: https://doi.org/10.64898/2026.01.09.698728; this version posted January 12, 2026. The copyright holder for this preprint (which was not certified by peer review) is the author/funder, who has granted bioRxiv a license to display the preprint in perpetuity. It is made available under aCC-BY 4.0 International license.

Supplementary Figure 9

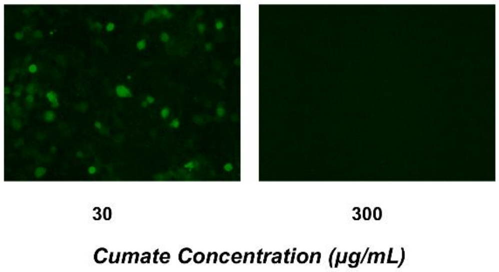

Supplement: Supplement 1 — Supplementary Figure 1. Flow cytometry analysis comparing HA surface expression using a commercial PiggyBac transposase and a non-commercial engineered PiggyBac transposase. Supplementary Figure 2. Flow cytometry analysis comparing HA surface expression in constructs expressed with or without the GCN4 trimerization motif. Supplementary Figure 3. HA surface expression following PiggyBac mediated genomic integration versus transient transfection Supplementary Figure 4. Flow cytometry analysis comparing HA surface expression in constructs lacking or containing an IRES–GFP expression cassette. Supplementary Figure 5. Flow cytometry analysis showing the effect of varying total DNA amounts on HA surface expression. Supplementary Figure 6. Flow cytometry analysis showing the effect of varying puromycin concentrations on selecting for HA expressing cells. Supplementary Figure 7. Antibody titration analysis of the enriched HA libraries following the second round of sorting, showing concentration dependent binding to the head-interface antibodies FluA20 and S5V2–29. Supplementary Figure 8. Flow cytometry analysis of GFP and mCherry expression showing reduced GFP+ mCherry double positive populations following dilution with the carrier transposon. Supplementary Figure 9. Fluorescence microscopy images showing HA and -GFP co0-expression following induction with 30 μg/mL cumate and complete cell death following induction with 300 μg/mL cumate. [file NIHPP2026.01.09.698728v2-supplement-1.pdf]
